# Frailty: A Nebulous Concept?

**DOI:** 10.1016/j.shj.2024.100337

**Published:** 2024-07-15

**Authors:** Anthony DeMaria

**Affiliations:** Judy and Jack White Chair in Cardiology, Sulpizio Cardiovascular Center, University of California, San Diego, La Jolla, California

The existence of frailty and its importance to overall health have almost certainly been recognized since the beginning of time. However, the exact definition and precise criteria for this condition continue to be unsettled. I remember many, many years ago, being a medical student on my first clinical rotation and hearing the residents and interns refer to a condition called “PPP.” Being unable to find this condition described in textbooks, I was finally told that the letters stood for “piss-poor protoplasm.” The term was meant to convey a patient whose general state was characterized by widespread deterioration of functions, high risk for invasive procedures, and a poor prognosis. When I asked how I could identify if a patient had frailty, I was told that it was akin to pornography; you know it when you see it. Although considerable progress has been made toward establishing the definition and quantification of criteria for frailty, in many respects, it continues to be a somewhat nebulous entity.

While doing some background reading on the topic recently, I learned that 2 distinct types of frailty have been described.^1^ Deficit accumulation frailty relates primarily to cumulative illnesses, while physical frailty largely relates to the effects of aging. I must admit I found the deficit accumulation variety a bit confusing and sounding more like disability: did patients become ill because they were frail or were they frail as the result of illness? Physical frailty is the more clinically relevant type, and it is the one I focus on in this piece.

I was astounded by the number of definitions for frailty that I encountered in my research. The one I liked best took elements from several others and generalized. It described frailty related to aging as a state of decreased reserves resulting in increased vulnerability to adverse outcomes when exposed to stressors. I liked it because it conveyed the concept that a person could be frail without or prior to any illness. Although the etiology of frailty is uncertain, it is often ascribed to a dysregulation of endocrine, immune, and other stress response systems, possibly related to genetic and environmental factors. Clearly, that is colossally nonspecific.

While the definition and the etiology of frailty are at best vague, the diagnostic criteria to establish the presence of the condition are absolutely unsettled. A recent review listed 67 different diagnostic schemas, and those were only the ones that were said to be most cited.^2^ The most applied is the Fried Frailty Tool, which is comprised of 5 components: weight loss, sense of exhaustion, weakness (grip strength), slow walking speed, and decreased physical activity. Although many other diagnostic instruments incorporate these same characteristics, the method of assessment and the quantification often differ. One diagnostic algorithm utilizes the eponym “FRAIL” (fatigue, resistance, ambulation, illness, loss of weight) to screen for the condition, while another incorporates the ability to get up from a chair without using the arms. I have heard some physicians say that all they do to screen for frailty is have patients sit on the floor and see if they can get up. I can think of no condition in cardiovascular disease in which the criteria for diagnosis are so unresolved and open-ended.

A number of other issues contribute to the fuzziness of the frail state. Given the multiple definitions proposed, it is not surprising that there is a wide variation in the prevalence of frailty that has been reported. Several additional variables, including environmental, educational, psychological, and socioeconomic factors, can influence the appearance and expression of frailty. Although exercise training has been found to be of therapeutic benefit in addition to nutritional supplementation and cognitive training, it is unclear how patients who are exhausted, weak, and often sarcopenic can participate in meaningful physical conditioning.

The existence and level of frailty are assessed, directly or indirectly, in every patient by every physician. It is often expressed in terms of physiological vs. chronological age. It is extremely important in formulating therapy and estimating prognosis and can be the determining factor as to whether a patient is offered an invasive procedure or referred for palliative therapy. Frailty is especially important in structural heart disease since such a high percentage of our patients are elderly. It is astounding, therefore, that such a critically important condition is so nebulous. The definition is general and relatively nonspecific, the diagnostic criteria are largely qualitative and unsettled, the distinction from comorbidities is often difficult or impossible, and the role of environmental and socioeconomic factors is often uncertain. After some forty-plus years since my first encounter with PPP, the identification of frailty continues in some respects to be similar to that of pornography. In my view, frailty is one of the medical conditions that most cries out for research and standardization.
